# Toward In Situ Stabilization of Raw Chinese Lacquer (*Toxicodendron vernicifluum*): Current Evidence, Processing Strategies, and Research Challenges

**DOI:** 10.3390/polym18162028

**Published:** 2026-08-21

**Authors:** Ziyue Zhang, Baoju Jin, Xiaotong Li, Hanyun Gao, Xinhao Feng

**Affiliations:** 1 College of Furnishing and Industrial Design, Nanjing Forestry University, Nanjing 210037, China; zhangziyue@njfu.edu.cn (Z.Z.); lixiaotong@njfu.edu.cn (X.L.); ghy0128@njfu.edu.cn (H.G.); 2 Co-Innovation Center of Efficient Processing and Utilization of Forest Resources, Nanjing Forestry University, Nanjing 210037, China

**Keywords:** raw lacquer, in situ processing, review methodology, filtration, vacuum dehydration, quality regulation, enzymatic activity

## Abstract

Raw Chinese lacquer, tapped from the sap of *Toxicodendron vernicifluum*, is a natural water-in-oil microemulsion containing urushiol, polysaccharides, proteins, and laccase. Because this reactive system continues to oxidize and polymerize after harvesting, handling conditions directly determine water content, viscosity, and later film-forming performance. This review analyzes potential in situ stabilization routes that couple purification, low-temperature vacuum dehydration, and quality conditioning at, or near, the collection site. Emphasis is placed on how laccase retention, oxygen exposure, and urushiol polymerization are controlled together to limit transport losses and premature crusting. Portable filtration devices, reported centrifugal filtration systems, and proposed vacuum dehydration strategies are compared in terms of throughput, field compatibility, and process control. Physical and bio-based conditioning strategies, including shear adjustment, oxygen management, and natural film-forming aids, are further considered for on-site regulation. Surface-enhanced Raman spectroscopy (SERS) and portable spectroscopic devices are examined as feedback tools for parameter adjustment under field temperatures, humidity, and storage variation; however, these signals are treated as decision-support indicators that still require lacquer-specific calibration after tapping. The central task is to define a field-compatible process window for water removal, laccase retention, viscosity control, drying behavior, and storage stability before downstream coating preparation. The remaining challenges involve miniaturized equipment, standardized evaluation, evidence-level classification, and dynamic control of coupled variables.

## 1. Introduction

Raw Chinese lacquer, obtained from the phloem of *Toxicodendron vernicifluum*, is a solvent-free, water-in-oil (W/O) microemulsion composed mainly of urushiol, water, polysaccharides, glycoproteins, and laccase [1,2,3]. Its processing difficulty arises before film formation: once exposed to air after tapping, impurity entry, water migration, oxygen diffusion, laccase-catalyzed oxidation, and urushiol coupling proceed at the same time. These coupled changes quickly alter viscosity, oxygen exposure history, storage stability, and later drying behavior in the collected sap, even before formal refining actually begins during early handling stages.

Moving the processing forward is required because harvest-induced contamination and chemical reactivity begin together. Bark fragments, dust, and insects enter the sap during tapping; the bark’s anatomy explains why plant debris is difficult to avoid [1]. Primary purification studies likewise indicate that contamination control must start in the field [2]. Portable-device and porous-media filtration studies further show that impurity removal must be balanced against portability and material compatibility [3,4]. At the same time, laccase-catalyzed oxidation converts urushiol into dimers, trimers, and oligomeric networks that support film formation but also drive crusting and viscosity increases during storage [5,6]. Spectroscopic and hardening studies connect this polymerization state to later film behavior [7,8]. Because urushiol is also sensitizing, longer open handling increases both exposure risk and material loss [9,10]. Traditional refining addresses part of these changes; however, much of the water removal, stirring, and oxidation control still occurs only after transport and before sealed stabilization begins [11,12,13,14].

Accordingly, the critical issue in raw lacquer harvesting and pre-treatment is not efficiency in any single step, but how soon purification, dehydration, and initial conditioning can be completed before quality decay develops. Shifting filtration, concentration, and quality regulation to the harvest site or nearby points shortens the interval for polymerization during transport and storage, lowers the exposure risk, and delivers consistent raw material for later refining.

**Figure 1:**
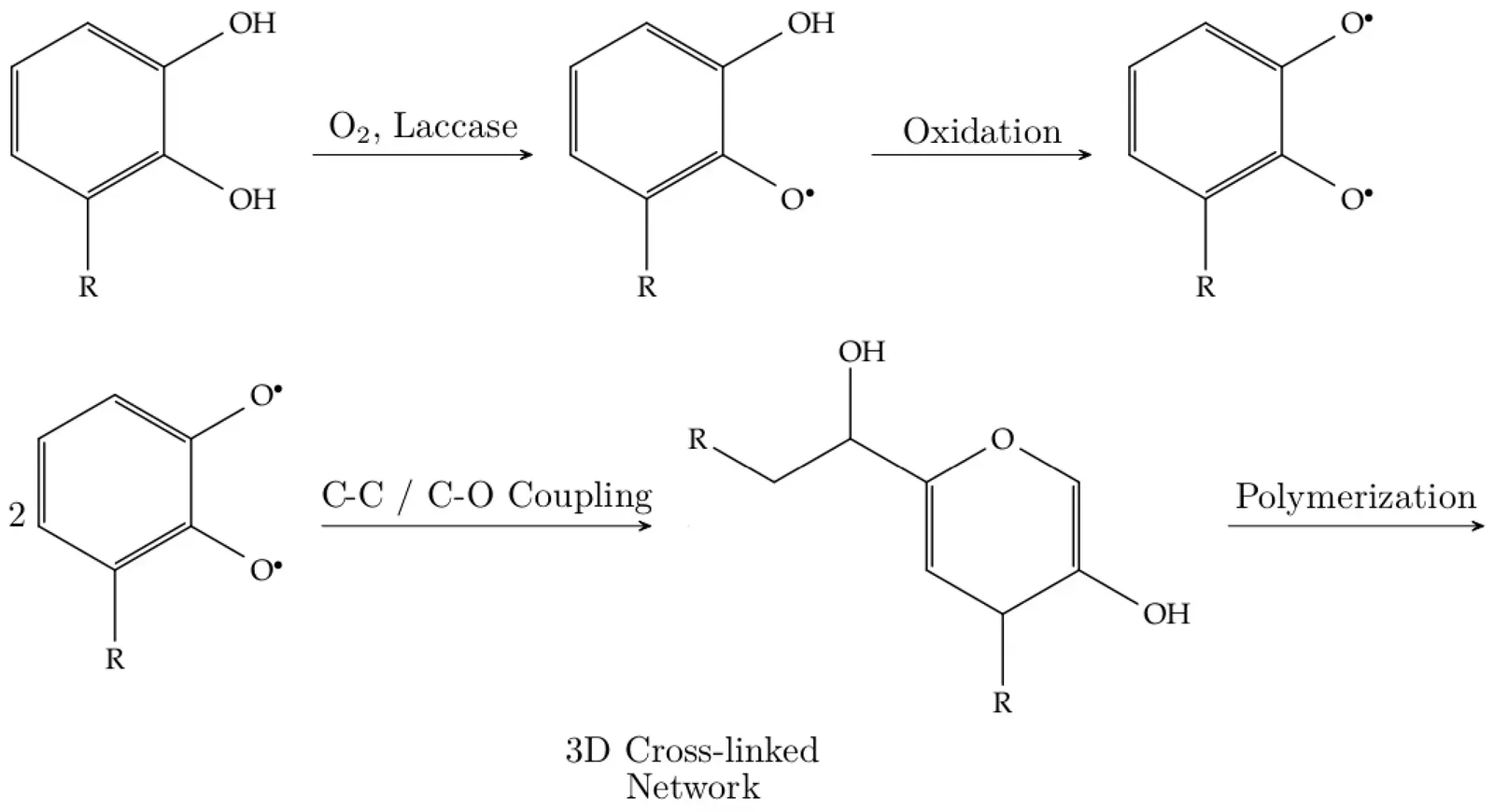
Core oxidation and polymerization pathway of urushiol during raw lacquer film formation.

Figure 1 uses urushiol oxidation, free-radical generation, coupling, and 3D network growth to define the mechanism discussed in this review. The scheme shows that post-harvest deterioration of raw lacquer after tapping is driven by simultaneous enzymatic oxidation, side-chain auto-oxidation, and polymer network development, rather than by moisture loss alone. Filtration, dehydration, and conditioning should, therefore, be judged by how effectively this slow uncontrolled change while retaining film-forming performance during field handling.

**Figure 2:**
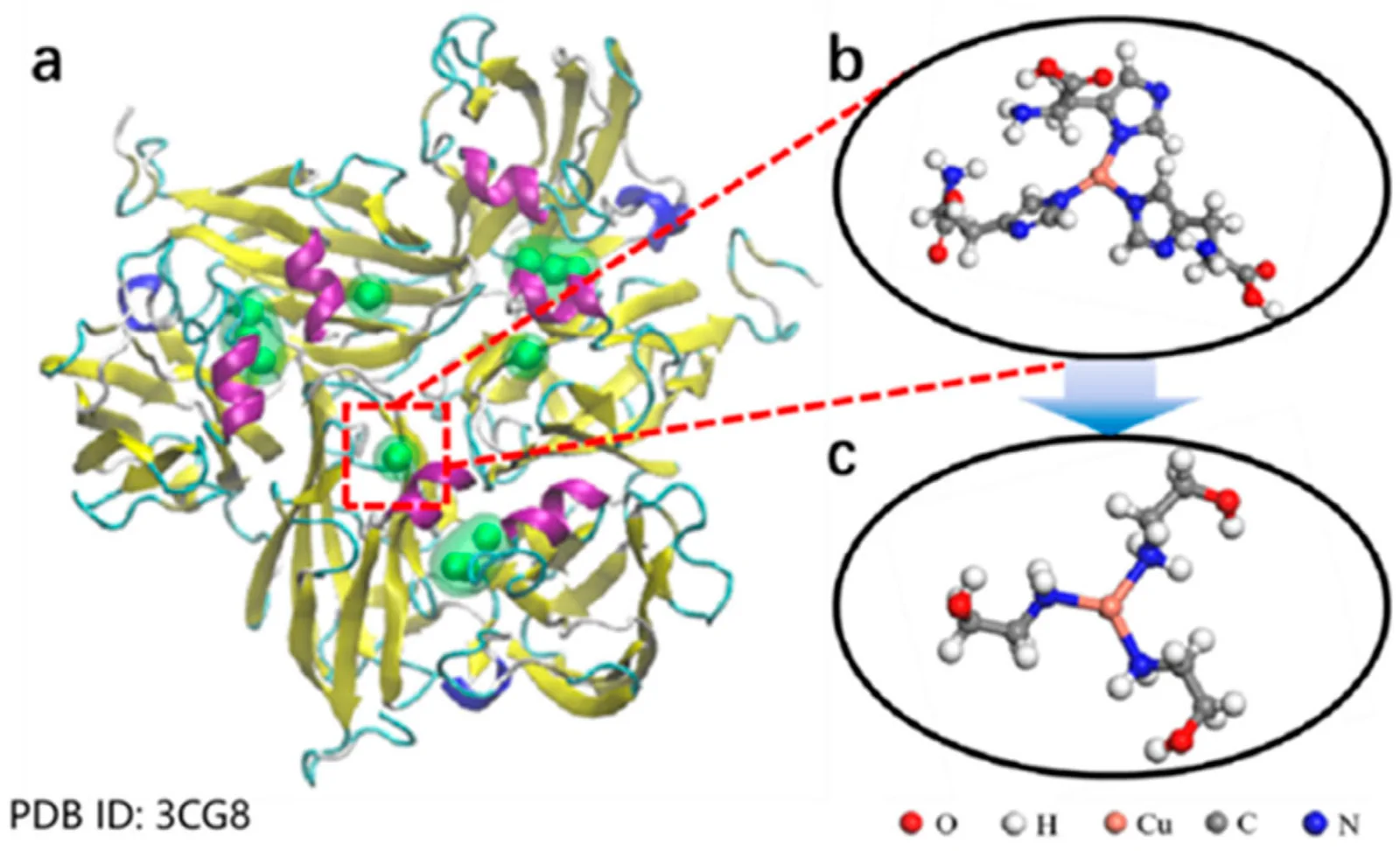
Structural representation of laccase and its active-site region relevant to urushiol oxidation in raw lacquer: (**a**) overall laccase protein structure based on PDB ID 3CG8, with different colors used to distinguish protein secondary-structure elements and highlighted regions; (**b**) enlarged view of the active-site molecular structure; (**c**) further detailed view of the copper-associated molecular structure. O, H, Cu, C, and N atoms are indicated by red, white, pink, gray, and blue spheres, respectively [5].

This front-loaded processing logic follows directly from the laccase-centered curing pathway. Figure 2, therefore, emphasizes the three-dimensional laccase structure and its copper-containing active region, because these features determine how processing conditions affect urushiol oxidation.

Seen from this structural perspective, raw lacquer processing cannot be judged only by impurity removal or final water content. Filtration, dehydration, and conditioning must limit contamination and transport loss while keeping temperature, shear, pH, and oxygen exposure within a range that preserves laccase activity and stable urushiol oxidation.

This enzyme-based view frames the industrial problem addressed here. Under the traditional dispersed harvesting-centralized processing model, sap continues to change chemically and physically during transport and storage, making processing efficiency and product consistency difficult to control. In situ processing, therefore, seeks to complete purification, dehydration, and preliminary conditioning near sap collection so that active components are retained and downstream refining starts from a more stable feedstock before shipment.

For raw lacquer, in situ processing changes the order of operations because fresh sap changes rapidly after harvesting. Green pre-treatment studies for natural products point in the same direction: low sample volume, low solvent consumption, and rapid handling are most effective when reaction-prone materials are processed immediately [2]. Related natural-product processing studies provide methodological references for low-temperature and rapid stabilization; however, their parameters cannot be directly transferred to raw lacquer because of its reactive W/O microemulsion structure.

From the viewpoint of green manufacturing and supply-chain design, in situ processing at the production site is not defined by isolated equipment alone. Material flow, energy use, data capture, and quality traceability must also be coordinated. In this sense, in situ processing is a system-level reorganization linking harvesting, mobile units, detection modules, and downstream refining.

Existing reviews on raw lacquer largely focus on chemical composition, laccase-curing mechanisms, film properties, and refining/modification processes, with insufficient attention to maintaining sap quality immediately after harvesting at the production site. English-language studies in the literature on Eastern lacquer chemistry, East Asian lacquer conservation, thermal characterization of films, and raw lacquer modification applications provide a foundation for establishing a “composition–reaction–processing–performance” analytical framework. Unlike prior studies, this review emphasizes process front-loading and on-site processing, integrating filtration, dehydration, conditioning, and on-site detection within a single technical framework to assess their potential integration and identify validation requirements for the rapid and stabilized post-harvest treatment of raw lacquer [15]. The differences in research scope and the specific contribution of this review compared with previous lacquer-related reviews are summarized in Table 1. Importantly, this review does not consider in situ processing as an established industrial solution; rather, it evaluates the current evidence base, technological limitations, and future validation requirements associated with this emerging concept.

This article was revised as a critical narrative review rather than a systematic meta-analysis. To make the review process reproducible, the literature corpus was reconstructed from the cited references and targeted searches in Web of Science, Scopus, Google Scholar, CNKI, and publisher databases. Search terms combined material names and process terms, including raw Chinese lacquer, *Toxicodendron vernicifluum*, urushi, lacquer sap, laccase, urushiol, filtration, vacuum dehydration, low-temperature refining, in situ processing, portable detection, Raman, SERS, and field quality control. The searched publication window covered foundational lacquer-enzyme studies from 19 onward and recent process-engineering and detection studies through to 2026. English and Chinese sources were considered when they addressed raw lacquer chemistry, refining, post-harvest quality, filtration, dehydration, monitoring, or closely related transport principles. Studies were excluded from direct evidence claims when they concerned unrelated food, membrane, slurry, sensor, or natural-product systems without raw lacquer validation; these sources were retained only as conceptual or engineering analogues. The final cited corpus, therefore, includes lacquer-specific experimental studies, equipment or patent-oriented studies, conservation and coating reviews, and carefully labeled cross-domain analogues. The composition of the reviewed literature corpus and the classification of evidence sources used throughout this review are summarized in Table 2.

## 2. In Situ Processing System Framework: Purification, Dehydration, Conditioning, and Closed-Loop Monitoring

### 2.1. Concept and Core Elements of In Situ Processing

The concept of in situ processing originated in the mining and petroleum industries, emphasizing the completion of processing or treatment with minimal raw material transfer. Recently, this concept has been introduced into natural product processing, referring primarily to purification, dehydration, and preliminary conditioning at, or near, the collection site. In this review, in situ processing is considered as an early-stage stabilization strategy performed at, or near, the harvesting site before centralized refining. It is intended to reduce quality variations during handling and transport rather than to replace conventional refining or produce application-ready lacquer directly.

Operationally, in situ processing in this review means a field-adjacent stabilization workflow performed at the tapping site or nearby collection point before long-distance transport or centralized refining. It is not defined as a complete substitute for all traditional refining steps. Its minimum target is to remove coarse impurities, reduce uncontrolled water migration and oxygen exposure, slow viscosity drift and crust formation, and preserve laccase-mediated curing capacity. The intended system is, therefore, portable or modular, able to operate on small, dispersed harvest batches, and designed to deliver a stabilized feedstock for downstream refining rather than guaranteed application-ready lacquer.

Purification (impurity removal) involves eliminating solid impurities and coarse particles from lacquer sap to obtain a clean base material. This stage directly affects the efficiency of subsequent refining and the quality of lacquer films. Sap duct distribution, diameter, and density in the bark cross-section vary significantly among cultivars [1], meaning that impurity loads differ by tree type and require tailored purification strategies. Comparative studies of bark structure, sap yield, and urushiol content further confirm that duct diameter and bark thickness are positively correlated with sap yield, providing a scientific basis for cultivar-specific parameter settings in in situ processing [1].

Dehydration (concentration) aims to reduce water content while protecting laccase activity, keeping water content, viscosity, and oxidation within a window suitable for subsequent application. Water content directly influences microdomains in the W/O microemulsion and the formation of lacquer films [16]. Laccase preservation is equally important because enzyme activity governs the oxidation pathway of urushiol [5]. Equipment-based refining studies show that moisture removal, stirring, and temperature must be managed as coupled process variables rather than isolated operations [12]. Low-temperature vacuum drying of plant materials emphasizes the role of the vacuum degree [17], while related dehydration work highlights temperature and quality retention [18]. Thin-layer drying clarifies the importance of mass-transfer path length [19], and response-surface optimization studies show that residence time and process parameters should be modeled together [20,21]. Consequently, miniaturization, sealing, and precise parameter control of in situ dehydration equipment are essential for reducing the transport burden and improving field processing efficiency.

Conditioning (quality regulation) refers to the physical or chemical modifications used to adjust drying behavior, rheology, and color. In raw lacquer, refining also involves a biochemical transition because the laccase-catalyzed oxidation of urushiol drives dimer and polymer formation and thereby changes curing behavior [5,12]. Urushiol, a mixture of catechol derivatives with saturated, mono-, di-, and tri-unsaturated side chains, forms the main film skeleton and also varies with region and cultivar [22]. Lacquer tree laccase is a copper-containing polyphenol oxidase; its conserved metal-binding site helps to explain why oxidation control remains central during in situ processing [23]. Microcalorimetry can quantify Michaelis constants and enzyme activity, providing one possible route for the on-site assessment of laccase activity during in situ processing [24].

Taken together, purification, dehydration, and conditioning function as one continuous chain for stabilizing sap quality. Impurity removal reduces the load entering later steps, dehydration controls water content while preserving microemulsion structure and laccase activity; conditioning sets viscosity, drying behavior, and film-forming performance. The value of in situ processing, therefore, depends on linking these operations in sequence so that post-harvest change is controlled before it becomes irreversible.

To avoid overstating technology maturity, the following sections distinguish three evidence levels based on the evidence categories summarized in Table 2: demonstrated raw lacquer evidence; laboratory-scale processing evidence that requires scale-up; and cross-domain concepts transferred from food, membrane, slurry, sensor, or natural-product studies. Technologies in the third group are discussed as hypotheses or design inspirations unless lacquer-specific compatibility, fouling, laccase retention, and coating-performance data are available.

### 2.2. Potential Benefits and Remaining Limitations of In Situ Stabilization

When purification, dehydration, and conditioning are linked at the harvest site, fresh sap spends less time exposed to air and the risk of laccase inactivation falls. The location shift first reduces transport volume and, after early water removal, the weight carried downstream. This also narrows the impurity burden entering later refining stages. The shorter delay before treatment lowers enzyme activity loss and polymerization-induced crusting during storage and transit. Downstream refining, therefore, starts from sap with a narrower range of impurity load, water content, and viscosity, which improves raw lacquer uniformity and supports higher-value products.

As shown in Table 3, the main advantage of in situ processing is not only the shorter processing time, but earlier control of the variables that change during transport and storage. In centralized processing, sap continues to undergo enzyme inactivation, oxidative polymerization, and impurity accumulation before refining begins; thus, later steps must compensate for earlier deterioration. By moving purification, dehydration, and preliminary conditioning to the collection site as early as possible, in situ processing reduces that degradation window and supplies downstream standardized refining with a more stable raw lacquer input.

### 2.3. Feedback-Assisted Processing Framework for In Situ Processing

Miniaturizing conventional refining equipment is only one requirement; by itself, it cannot address the rapid post-harvest changes in raw lacquer. After tapping, impurity contamination, water migration, laccase-catalyzed oxidation, and viscosity increase proceed together; thus, optimizing one step in isolation does not ensure stable sap quality or downstream reproducibility in practice. Future in situ systems may connect harvesting, filtration, dehydration, conditioning, and on-site monitoring through a feedback-based workflow, although quantitative relationships between measured variables and process adjustments remain to be established.

**Figure 3:**
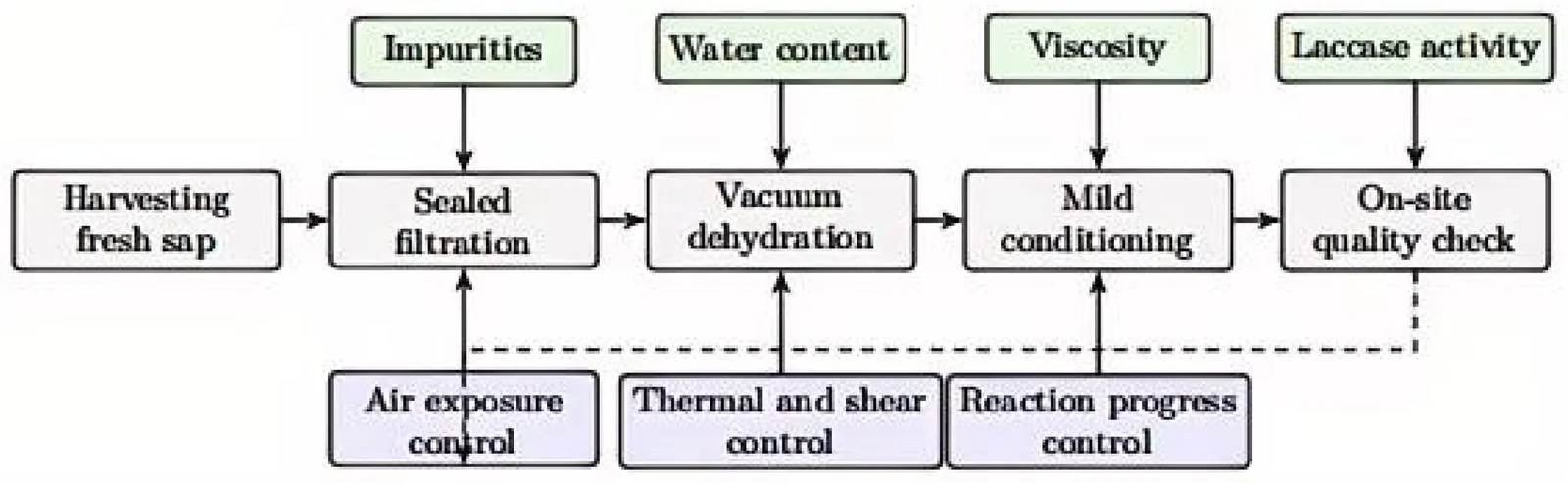
Conceptual feedback-assisted processing framework for in situ raw lacquer processing. White–grey boxes indicate the sequential processing stages, green boxes represent measurable quality parameters or feedback variables, and purple boxes indicate control or regulation actions used for process adjustment. Arrows indicate information feedback and process relationships rather than evidence of validated material flow.

Figure 3 assigns a different feedback variable to each stage: sealed filtration mainly reduces impurity load; vacuum dehydration tracks water content and temperature as the dehydration endpoint approaches; and gentle conditioning adjusts viscosity, oxidation levels, and reaction progress before film preparation. On-site quality detection then returns these measurements to upstream steps so that filtration, dehydration, and conditioning can be tuned as one connected control loop.

Within this framework, intelligent in situ processing needs to regulate impurity removal, water content, oxygen exposure, temperature, and stirring intensity as coupled variables. During refining, dehydration and oxygen uptake proceed at the same time as laccase-catalyzed reactions [6,17]. Studies on urushiol coupling and equipment-based refining likewise show that shear and temperature management directly affect reaction control [7,12]. The practical question is, therefore, how to maintain process efficiency without disrupting the enzyme-driven, film-forming system during mobile processing.

Oxygen management should also be stage-specific. During filtration and temporary storage, reduced air contact is desirable to limit premature surface oxidation and crust formation. During dehydration and conditioning, however, a controlled rather than oxygen-free environment is needed because laccase-mediated urushiol oxidation contributes to later curing. During final film formation, sufficient oxygen and humidity must be restored. This distinction reconciles storage stabilization with enzymatic curing requirements. These stage-specific control requirements are summarized as a conceptual decision logic for future in situ process optimization in Table 4.

**Figure 4:**
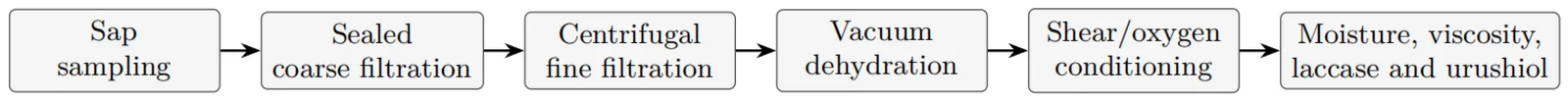
Author-generated workflow and key control point for in situ raw lacquer processing from sap sampling to sealed filtration, vacuum dehydration, mild conditioning, and on-site quality checks; it complements Figure 3 by separating operational steps from feedback variables.

The corresponding workflow and key control points for this integrated in situ processing chain are shown in Figure 4.

### 2.4. Relationship Between In Situ Lacquer Processing and Natural Product Processing Technologies

From a methodological perspective, related natural-product processing studies provide limited references for designing low-temperature and rapid stabilization strategies. However, these studies do not represent direct evidence for raw lacquer processing because the preservation of laccase activity, microemulsion structure, and coating performance imposes specific constraints [2]. Studies involving ultrasound-assisted extraction, microwave treatment, and other mild processing approaches suggest general principles of reducing thermal or processing stress; however, their parameters cannot be directly transferred to raw lacquer systems [25,26]. Non-thermal starch modification and plant-extract processing studies provide additional examples of targeted regulation under mild conditions [27,28]. For lacquer-specific raw materials, ultrasonic extraction of leaf urushiol and natural bioactive extraction studies indicate that selective processing must be matched to the substrate chemistry rather than copied mechanically [29,30]. These general principles are consistent with some design considerations for raw lacquer in situ stabilization; however, their applicability requires lacquer-specific validation [31].

**Figure 5:**
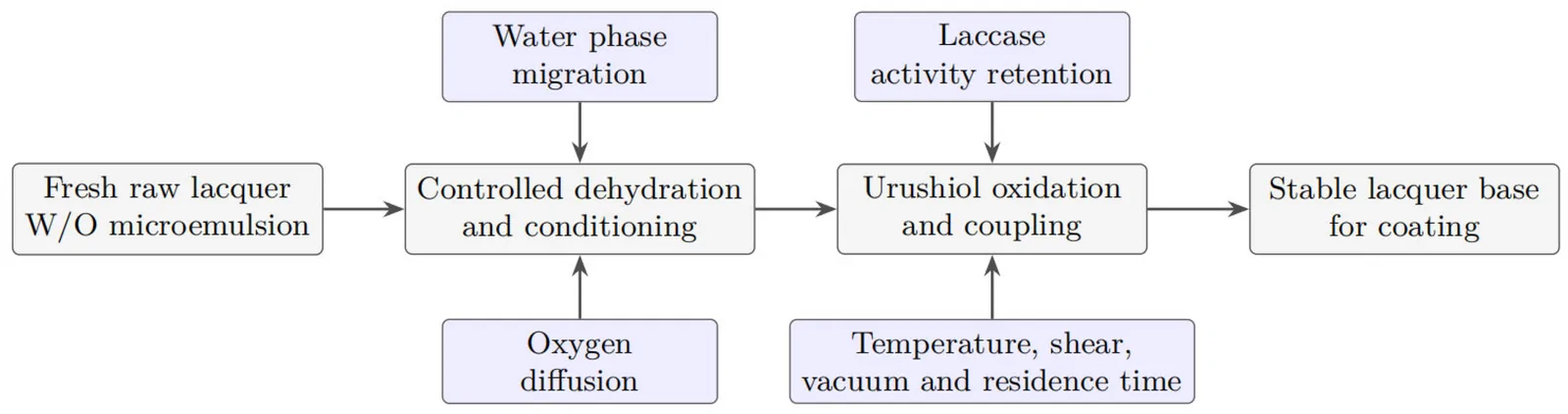
Conceptual mechanism illustrating the relationship between dehydration, enzymatic oxidation, and film-forming potential in raw lacquer stabilization. In this figure, colored boxes represent influencing factors and mechanistic variables rather than feedback-control parameters. Unlike Figure 3, Figure 5 focuses on process mechanisms instead of closed-loop regulation.

To illustrate this distinction, Figure 5 links dehydration, enzymatic oxidation, and film-forming potential. Unlike Figure 3, which emphasizes feedback-based process regulation, Figure 5 focuses on mechanistic relationships among influencing factors and does not represent an operational control loop. For raw lacquer, in situ processing must control water removal and impurity reduction without pushing urushiol oxidation beyond the range needed for later film formation. These limits are set by laccase retention, viscosity growth, and drying performance. The same treatment that improves storage stability can also lower laccase activity and disturb W/O microemulsion structure if vacuum, temperature, or residence times become excessive. Unlike extraction-oriented natural-product processing, raw lacquer treatment, therefore, aims to preserve reaction capacity and define a workable parameter window rather than to stop reactions completely.

## 3. Evidence-Based Purification Technologies for Raw Lacquer Stabilization

### 3.1. Limitations of Conventional Filtration and Innovations in Portable Devices

Filtration is the first operation in raw lacquer processing and strongly influences both the subsequent refining efficiency and final product quality. Studies on green sample pre-treatment and on-site sensing indicate that field equipment must be lightweight, rapid, and easy to maintain [2,32]. Work on dynamic membranes and cartridge purification further shows that separation precision must be balanced against fouling control and media regeneration [3,33], while quick, easy, cheap, effective, rugged, and safe (QuEChERS)-type pretreatment illustrates the value of simple modular purification under complex sample conditions [4]. Anatomical studies of lacquer bark explain why tapping inevitably introduces bark fragments from sap ducts in the secondary phloem [1]. Traditional field filtration usually relies on gauze or a filter cloth under gravity, which is slow and ineffective for fine impurities. Industrial centrifuge filtration increases impurity removal; however, the gain in impurity removal is accompanied by increases in equipment volume, power demand, and operational complexity that do not fit dispersed tapping sites.

To match the needs of lacquer farmers and artisans for low-cost, low-effort, safe, and easy-to-use devices, several portable filtration formats have been developed. Hand-crank units combine a base, clamps, rollers, and filter mesh so that one operator can complete filtration. Stirred-box devices use motor-driven rods and brushes to limit residue buildup and enable quick replacement of the filter element. Pressure-assisted systems push sap through the filter plate and raise throughput markedly. At the material level, ultra-high molecular-weight polyethylene (UHMWPE) filter media have shown potential advantages for automated filtration and media regeneration in other liquid separation applications [3]. UHMWPE tubes exhibit characteristics such as 35–42% porosity, 5–50 μm filtration precision, corrosion resistance, self-lubrication, and high backwash regeneration efficiency [4]. However, these properties cannot be directly interpreted as evidence for raw lacquer filtration performance. Their applicability to in situ raw lacquer purification requires further evaluation that considers fouling behavior, interaction with urushiol components, preservation of laccase activity, and influence on final film properties.

Recent developments in two-dimensional nanosheet membranes also provide insights for raw lacquer filtration. Materials, such as graphene oxide (GO), MXene, MoS_2_, and 2D metal–organic frameworks (MOFs), can form layered sieving channels for fine-particle retention and selective mass transfer [3]. However, these membranes are mainly suitable for low-viscosity aqueous systems; direct application to raw lacquer faces challenges such as pore blockage, urushiol adsorption, cleaning, regeneration, and safety concerns. Their value lies not in the immediate replacement of gauze or filter cloth but in guiding future filter design to balance pore control, anti-fouling surfaces, non-metal contact, and minimal interference with laccase activity. Therefore, these materials should currently be regarded as exploratory candidates rather than validated raw lacquer filtration technologies.

### 3.2. Centrifugal Filtration: Engineering Progress and Validation Requirements

Building on portable filtration, in situ filtration technology has been proposed to advance toward high-efficiency, continuous operation. For viscous raw lacquer systems, future filtration designs should integrate sealed operation, pressure-assisted flow, and mechanical dispersion to improve throughput and reduce air exposure during purification. Positive-pressure centrifugal or sedimentation-assisted units may provide cross-domain engineering references for addressing several limitations of gravity filtration, particularly low throughput and filter clogging [33,34]. However, independent evaluation remains necessary regarding scalability, cleaning procedures, fouling behavior, and the preservation of lacquer properties after filtration.

**Figure 6:**
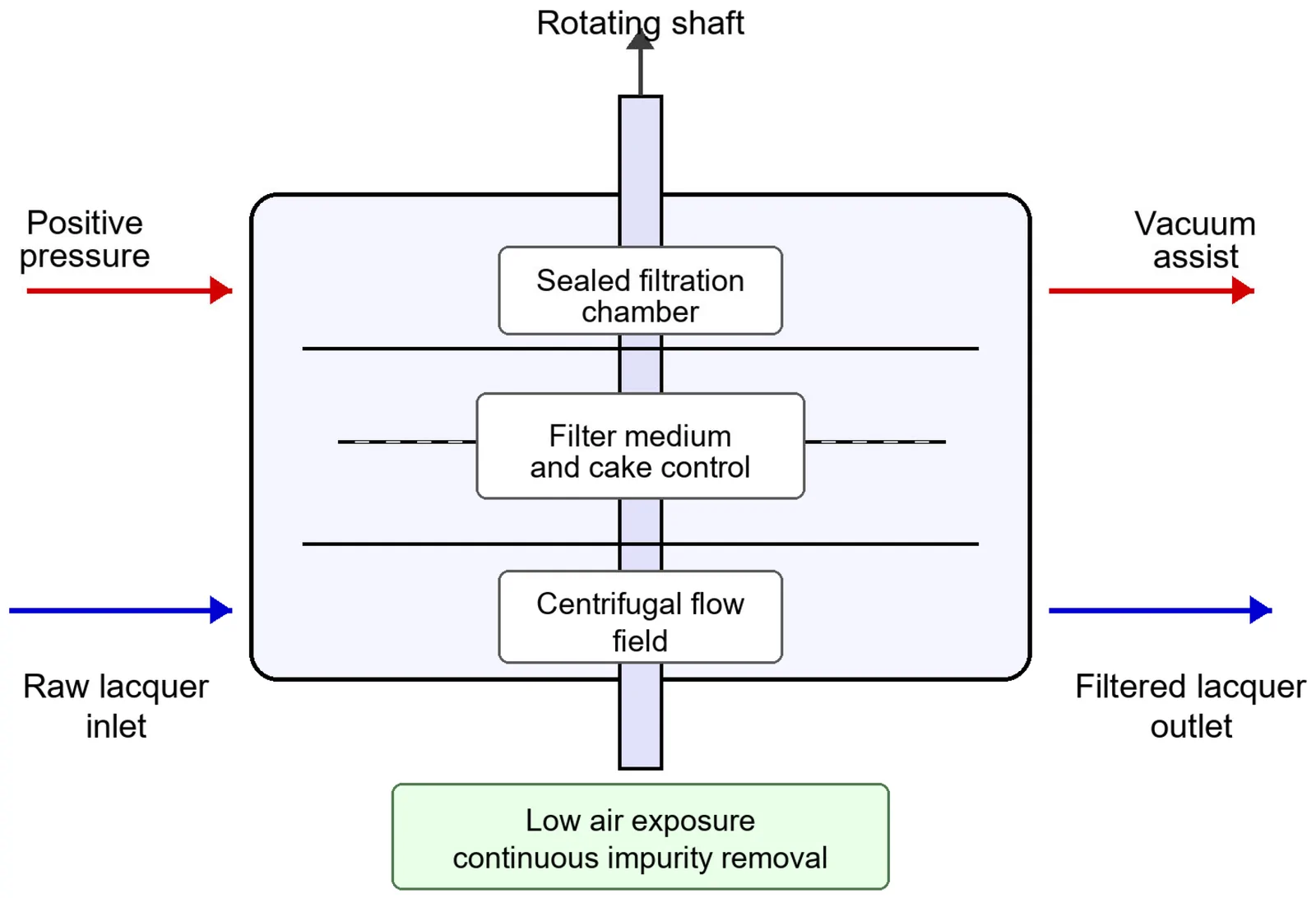
Positive-pressure centrifugal filtration unit for sealed and continuous purification, highlighting pressure–vacuum coupling, centrifugal flow, and low air exposure as the engineering logic for raw lacquer pre-cleaning.

Key innovations of the system include: (i) pressure coupling on both sides of the filter mesh to generate a significant differential, enabling rapid flow even for high-viscosity sap; (ii) centrifugal-induced rotational flow within the chamber to suppress rapid filter-cake accumulation and extend continuous operation time; and (iii) a sealed chamber reducing air contact, partially mitigating crust formation during filtration. Figure 6 summarizes this engineering logic as a continuous purification path based on pressure-difference driving, centrifugal cake control, and sealed low-exposure operation. Compared to traditional gravity filtration, this equipment better meets engineering requirements for rapid, sealed, and continuous in situ processing.

Independent assessment is still needed before this device can be treated as a validated field technology. Future reports should specify whether the system was experimentally tested, patented, modeled, or only conceptually designed, and should provide sample volume, pressure, centrifugal speed, processing time, filtrate recovery, cleaning procedure, fouling rate, and post-filtration lacquer properties.

The application of annular centrifugal extractors in continuous nanoparticle separation provides inspiration for continuous device design [33]. Inclined-settler and oil-absorption purification studies show that viscous or particulate-containing liquids require careful control of flow-channel structure and interface enrichment [4,34]. Given raw lacquer’s complex water-in-oil microemulsion and the sensitivity of laccase to solvents and interfacial conditions, such liquid-liquid extraction concepts cannot be directly transferred. Their value lies in suggesting sealed swirling flow, continuous feed/withdrawal, and controlled impurity migration paths without introducing new extraction chemistry.

### 3.3. Existing Challenges and Future Directions

Portable filtration has progressed; however, field use in raw lacquer still depends on three coupled constraints. Finer meshes remove more impurities but reduce throughput. Filter media compatibility is equally critical, because metal contact can alter urushiol chemistry or introduce ions that change sap color and laccase activity [16,35]. Air exposure during filtration must also be limited, since faster separation can accelerate surface crusting. This means that flux data alone are inadequate for process selection in the field. Filtration performance should, therefore, be judged not only by separation efficiency, but also by sap color, laccase activity, viscosity change, and the film-forming behavior of the treated lacquer.

**Figure 7:**
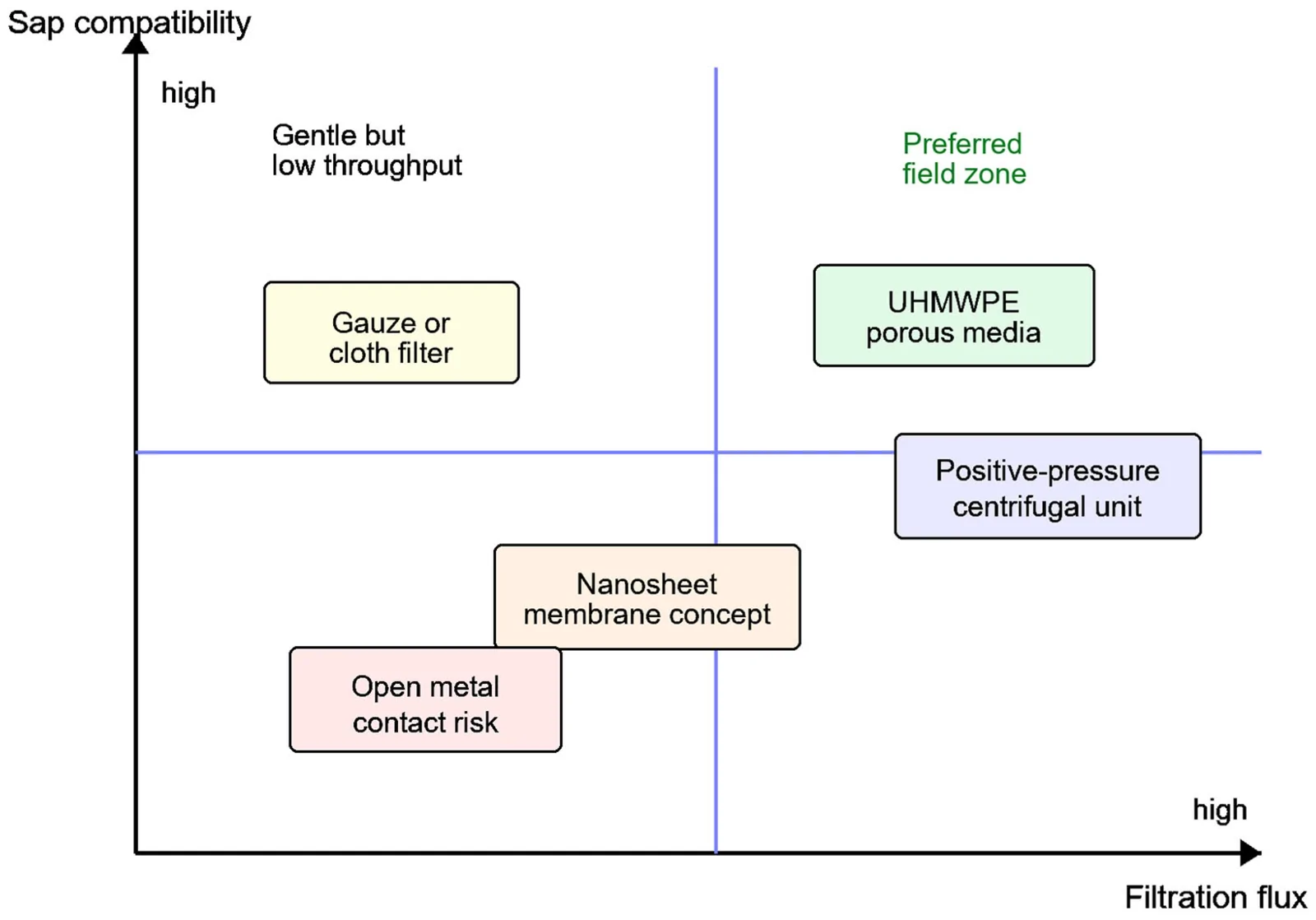
Filtration technology map for matching separation efficiency with raw lacquer compatibility.

For field deployment, in situ filtration devices should emphasize three design targets: polymeric or composite filter media that reduce metal contamination; low-energy drive systems that suit dispersed harvest sites; and modular coupling of filtration with preliminary dehydration so that harvesting, filtering, and concentration can proceed continuously. These choices determine whether filtration can be sustained under limited power supply, intermittent harvesting conditions, and low maintenance demand in practice. Figure 7 likewise suggests that ranking technologies by flux alone is insufficient. Effective field adaptation requires a joint evaluation of separation efficiency, enzyme activity retention, raw lacquer compatibility, and device portability.

Additionally, the filtration stage should be coupled with on-site quality detection. Tube-based SERS sensors can support the rapid screening of urushiol-related signals [32]. Portable analytical instruments provide a separate route for the field detection of polymerization degree and residual impurities [36]. Given the raw lacquer’s dark color, high viscosity, and complex composition, in situ spectral signals are easily affected by matrix interference. These tools are, therefore, best used for trend detection and anomaly alerts at the filtration outlet, combined with water content, viscosity, laccase activity, and film performance measurements to guide filter selection, media replacement, and subsequent dehydration settings.

Graphene oxide, MXene, MoS_2_, metal–organic framework, and other advanced membranes should, therefore, be described as prospective concepts rather than as established advances in raw lacquer filtration unless direct compatibility tests are available. Their assessment must include flux under lacquer viscosity, pressure differential, particle-retention range, adsorptive loss of urushiol or proteins, laccase retention, fouling rate, cleaning efficiency, energy demand, and possible chemical contamination.

Overall, purification represents one of the most established components of raw lacquer in situ stabilization. Conventional filtration remains practically relevant for impurity removal, while centrifugal and advanced membrane-based approaches provide opportunities for improving throughput and selectivity. However, the technological maturity of these approaches differs substantially. Future studies should prioritize raw lacquer-specific validation, including fouling behavior, laccase preservation, urushiol compatibility, cleaning procedures, and field-scale performance evaluation. The filtration approaches discussed above are compared in Table 5, which distinguishes established raw lacquer practices from exploratory cross-domain membrane and separation concepts.

Taken together, the filtration approaches compared above represent different evidence levels and maturity stages; Table 6 distinguishes established raw lacquer practices, reported lacquer equipment evidence, engineering evidence, and exploratory cross-domain membrane concepts according to their current maturity and validation requirements.

## 4. Low-Temperature Vacuum Dehydration: Water Migration, Laccase Activity Retention, and Process Window Optimization

### 4.1. Principles of Raw Lacquer Dehydration and Temperature Sensitivity

Fresh raw lacquer typically contains about 20–30% water, whereas refined lacquer for application and storage usually requires a much lower moisture range [5]. Reported values must be interpreted together with the sampling condition, film test method, and refining endpoint [5]. Temperatures of around 30–40 °C have been reported in lacquer refining studies; however, their suitability for in situ dehydration requires further evaluation because refining conditions do not necessarily represent field stabilization requirements. Pilot studies on lacquer refining indicate that temperature, stirring speed, and processing quality are the key factors affecting refining efficiency [12]. Within the biological temperature range, increases in temperature accelerate enzymatic reactions and shorten processing times; 30–40 °C is suitable for refining, while 60–150 rpm is generally favorable for oxygen transfer and water removal [5,12].

However, conventional heating presents two major drawbacks. First, processing cycles are long; traditional refining may require more than 12 h, limiting industrial scalability. Second, temperature control is challenging. Laccase, a copper-containing polyphenol oxidase, exhibits optimal activity between 20 and 30 °C and begins to inactivate above 40 °C; once inactivated, the sap irreversibly loses its natural film-forming capability. Purified laccase isoenzymes L1 and L2 exhibit optimal temperatures of 20 °C and 13 °C, respectively, underscoring the risk of enzymatic inactivation in heated dehydration [23,24]. Chemically, urushiol is a mixture of catechol derivatives with unsaturated side chains that undergo enzymatic oxidative polymerization [22]. Therefore, temperature control during dehydration directly affects the laccase retention and final film properties.

The apparent temperature window should be interpreted carefully: purified laccase optima do not necessarily equal the thermal tolerance of laccase embedded in the native W/O lacquer microemulsion; time-dependent inactivation may occur even when short exposure appears mild. Dehydration studies should, therefore, report both temperature and residence times when comparing rates of enzyme retention.

The enzymatic mechanism has been described in detail by Miyagwa and Kumanotani [6]: urushiol is oxidized by laccase to quinone radicals, which then undergo coupling reactions to form biphenyl dimers. Keilin and Mann clarified the oxidation-reduction behavior of laccase copper ions, explaining why laccase functions as a blue copper oxidase [37]. Later curing studies further showed that pH and alkaline conditions can affect laccase activity and film formation [35,38]. These studies indicate that the dehydration stage must control temperature and humidity without disturbing the enzyme-catalyzed redox cycle.

### 4.2. Principles and Advantages of Vacuum Low-Temperature Dehydration

Vacuum dehydration lowers the boiling point of water by reducing pressure so that water removal can proceed rapidly without the temperatures used in conventional heating. Its practical advantage in raw lacquer lies in coupling low-temperature evaporation with lower oxygen exposure, which helps to retain laccase activity, restrain excessive urushiol oxidation, and shorten the dehydration period.

Studies on agricultural drying provide transferable knowledge regarding vapor pressure reduction, mass transfer, and residence-time optimization. However, these studies mainly concern non-reactive biological materials and, therefore, cannot directly predict the behavior of raw lacquer, which contains an active enzyme system and reactive urushiol components.

Evidence transferred from food and agricultural drying is useful mainly for transport principles such as vapor-pressure reduction, thin-layer mass transfer, and residence-time optimization. It cannot, by itself, prove preservation of the urushiol microemulsion structure, laccase accessibility, or coating performance because raw lacquer is a reactive W/O enzymatic system rather than a plant tissue matrix.

**Figure 8:**
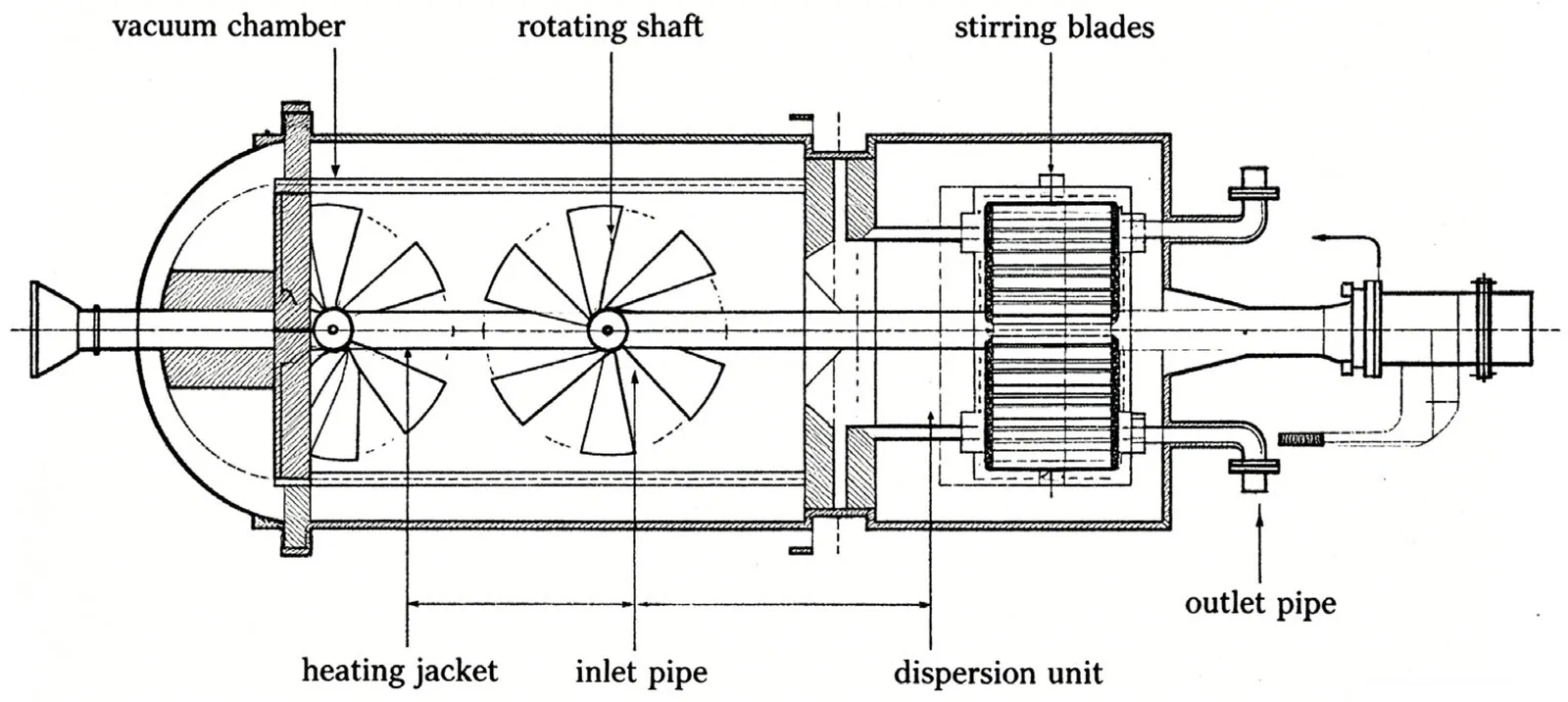
Conceptual representation of a vacuum-assisted lacquer refining device integrating sealed dehydration and shear-assisted processing.

Previous lacquer-processing studies indicate that low-temperature dehydration and mechanical dispersion may help shorten processing times while maintaining film-forming properties. In related device concepts, a sealed lid and vacuum interface create the low-pressure environment, the rotating shaft and Teflon scraping blades spread the viscous sap into a thin, moving layer, and the jacketed vessel provides controlled low-temperature heat transfer. Figure 8 should, therefore, be read as a coupled dehydration–shear device rather than as a simple vacuum pump. Its significance for in situ processing is that water removal, oxygen exposure, viscosity increase, and laccase activity can be managed in one closed chamber; however, the operating window must still be optimized to avoid overheating, excessive shear, or premature crust formation.

Reduced pressure may potentially influence droplet size distribution, phase behavior, dissolved oxygen balance, volatile components, interfacial protein organization, and enzyme accessibility in native lacquer systems. These potential structural effects require lacquer-specific investigation rather than an assumption that vacuum treatment acts only as a mild thermal operation.

The process window for vacuum dehydration is defined by the balance between water removal, laccase activity retention, and increases in viscosity.

Thin-layer processing shortens water migration paths but requires careful control of air exposure and shear to prevent accelerated urushiol oxidation. Compared to conventional heating-stirring methods, vacuum dehydration offers two major benefits: promoting water migration at a low temperature to reduce enzyme inactivation risk and significantly shortening refining cycles, thereby limiting polymerization losses during extended sap exposure. Importantly, the target is not simply minimum moisture content but an optimized parameter correlation among vacuum, temperature, residence time, solid content, and film performance parameters [39].

### 4.3. Effects of Refining on Drying Performance

Refining can shorten surface dry times; however, drying-time comparisons must distinguish between the surface dry time, practical dry time, and complete curing time. Experimental data show that when the solid content reaches approximately 80%, surface drying is slowest, although still faster than raw sap [5]. Yang et al. further showed that dehydration endpoint control affects gloss, viscosity, and workability during equipment-based refining [12]. This pattern can be explained by the coupled effects of oxygen diffusion, water content, and enzymatic polymerization: insufficient water removal limits handling, whereas excessive dehydration may reduce dissolved oxygen and delay surface drying. This provides theoretical guidance for controlling the endpoint of in situ dehydration.

For this reason, Table 7 should be read only as a conditional comparison unless film thickness, curing temperature, relative humidity, substrate, test method, and conditioning times are comparable across the reported materials.

### 4.4. Existing Challenges and Optimization Directions

Despite the advantages of vacuum low-temperature dehydration, in situ application faces several challenges: (1) equipment miniaturization and sealing reliability: most vacuum systems target centralized refining, and deployment at dispersed harvest sites encounters volume, energy, and maintenance issues; (2) precise endpoint control: overly low water content can prolong surface drying; endpoint solid content must match practical application performance; and (3) balancing enzyme activity retention with dehydration efficiency: excessive vacuum or shear may alter the sap microenvironment, affecting laccase activity and subsequent curing behavior.

For field dehydration, process evaluation should combine viscosity, water content, laccase activity, vacuum level, temperature, and shear intensity because these variables change together during water migration, microemulsion restructuring, and enzymatic polymerization. Unlike general slurries or food materials, raw lacquer also requires a dehydration rate, surface dry time, gloss, transparency, and storage stability as endpoint criteria. These outputs determine whether endpoint control remains usable during coating preparation. Practical development will require an evaluation of small-scale vacuum units, kinetic models for in situ processing, online water and viscosity monitoring, gradient vacuum, segmented temperature control, and low-shear stirring strategies. Future research should, therefore, focus on lacquer-specific kinetic models and field-scale validation rather than directly transferring parameters from conventional drying systems.

## 5. Quality Conditioning and Feedback Control for Early-Stage Raw Lacquer Stabilization

### 5.1. Principles and Advantages of Physical Modification

The physical modification of raw lacquer adjusts the rheology, drying behavior, and color without changing the chemical structure of urushiol in fresh sap after harvest. Because it works through shear, oxygen exposure, and natural film-forming aids rather than added reagents, it may help to maintain the native reactive characteristics of the sap under controlled processing conditions [13,40]. In practice, the goal is to tune viscosity, leveling, laccase-catalytic efficiency, drying, and gloss within a workable polymerization window, while keeping water content, oxygen exposure, and storage stability under control under field handling conditions.

Chemically, urushiol is a mixture of catechol derivatives—including saturated, mono-, di-, and tri-unsaturated species—that constitute the fundamental reactive components of lacquer films [22]. Physical modification can influence urushiol polymerization behavior and laccase activity by altering shear, oxygen exposure, and moisture distribution; however, quantitative control of these relationships remains insufficiently established. Studies on the curing mechanisms of air-drying oils indicate that oxidative polymerization proceeds through peroxide formation, decomposition, and cross-linking [41], which shares similarities with urushiol side-chain auto-oxidation. However, lacquer curing is also influenced by enzyme activity, water content, and humidity. Thus, in situ conditioning cannot simply adopt traditional drying oil strategies but must balance laccase catalysis and urushiol auto-oxidation, adjusting shear, oxygen exposure, moisture, and minimal additive incorporation.

### 5.2. Stirring Shear and Oxygen Control

Shear during stirring represents the most basic physical modification method. Pilot studies show that increasing stirring speed from 30 to 250 rpm can reduce refining time by approximately 70% [5]. Equipment-based refining studies further show that shear must be coordinated with vacuum dehydration and heat transfer [12]. However, excessive shear may disrupt the W/O microemulsion structure of the sap and compromise stability. When viscosity rises sharply with decreasing water content, high shear may also generate heat and accelerate laccase inactivation. Therefore, gradient-speed operation may represent a potential strategy for balancing water removal, microstructure preservation, and oxidation control. However, the relationship between shear rate, microemulsion stability, and final coating performance requires systematic validation.

The proposed boundary should be expressed experimentally by relating the shear rate or stirring speed to water removal, droplet structure, temperature rise, oxygen transfer, viscosity development, laccase activity, and film gloss/hardness. Until such datasets are available, the gradient–speed strategy remains a testable process hypothesis rather than an established operating rule.

### 5.3. Downstream Functional Modification and Its Boundary with In Situ Conditioning

Unlike early-stage conditioning, downstream functional modification mainly targets final coating performance rather than the immediate stabilization of freshly harvested lacquer. These approaches are, therefore, discussed as boundary technologies rather than core in situ processing operations.

Modification and performance enhancement provide the final layer of raw lacquer processing. Bio-based additives and nanomaterials may improve durability, color expression, or functional performance when their compatibility with lacquer chemistry is carefully controlled [42]. Cellulose and related natural polymers offer reinforcement routes with a lower environmental burden [43]. Epoxidized lacquer oil provides another bio-based strategy for changing flexibility and crosslinking behavior [13,44,45].

Chemical modification offers a more direct route for improving coating durability; however, it is less suitable for indiscriminate field addition. Epoxidized fatty acid hyperbranched resins can tune viscosity, hardness, and crosslinking density [46]. Environmentally friendly coating systems show a related route for durability improvement under a lower environmental burden [44]. For silane modification, Deng et al. used APTES to improve aging resistance and anticorrosive behavior [45]. Lu et al. separately showed that amino- and epoxy-functionalized silanes can accelerate polymerization under low humidity [14]. Precious metal nanoparticles may expand color and functional boundaries [47], while other bio-based or nano-reinforcement routes require strict control of dosage, enzyme compatibility, and long-term stability [42,43,48,49].

**Figure 9:**
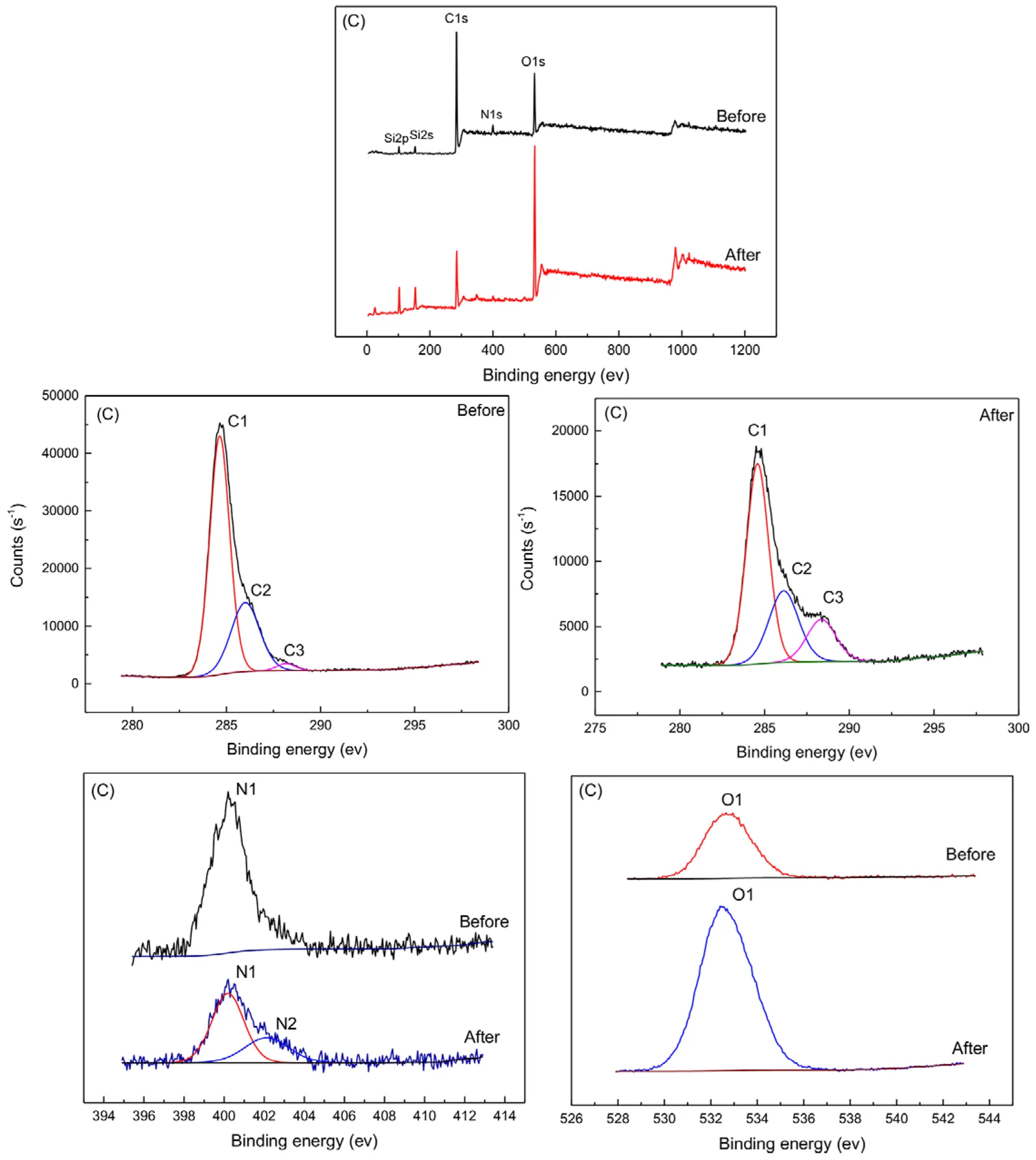
Example of downstream chemical modification affecting lacquer coating surface properties, illustrating the boundary between conditioning and application-stage formulation [45].

Figure 9 is used here to show how chemical modification alters the surface and bonding environment of lacquer-based coatings. The color spectral profiles indicate that modification should be assessed through chemical evidence together with gloss, hardness, film durability, and appearance. For in situ processing, chemical additives are, therefore, better treated as optional routes for targeted performance adjustment than as routine field-processing steps.

Accordingly, cellulose reinforcement, epoxidized oils, silanes, hyperbranched resins, and metal nanoparticles are treated here as downstream functional-modification options. They are relevant to in situ processing only when they clarify compatibility limits, storage stability, or the boundary between preliminary conditioning and application-stage formulation.

### 5.4. Chemical Conditioning Factors for Storage and Curing Balance

Inorganic salts and enzyme-related additives should be discussed as tightly controlled conditioning variables rather than routine additions. NaCl, Na_2_CO_3_, pH adjustment, and enzyme supplementation may influence water distribution, ionic strength, laccase activity, microemulsion stability, and urushiol oxidation through changes in the reaction environment and enzyme-mediated curing pathway [5,23,24]. However, their dosage windows remain uncertain for field processing and require lacquer-specific validation. Their use, therefore, requires measurements of viscosity, drying time, color, gloss, hardness, residual salts, corrosion risk, conservation compatibility, and long-term durability.

For storage-curing balance, the desired effect is not to suppress oxidation completely, but to delay premature crusting while retaining later enzymatic curing capacity through controlled regulation of oxidation and polymerization processes [5,6,22]. Any salt or enzyme strategy should, therefore, be evaluated under defined humidity, oxygen exposure, storage time, and film-curing conditions. Modern monitoring technologies may assist quality assessment during these conditioning processes by providing information on relevant physical and biochemical parameters; however, their field use also depends on portability, maintenance load, and process stability [50]. Process-monitoring sensors can record agitation, pressure, and vibration during field operations; however, these signals are meaningful only when interpreted alongside biochemical indicators such as laccase activity.

Therefore, conditioning strategies should currently be considered as compatibility-oriented regulation rather than established optimization methods. The effects of additives and environmental variables require systematic validation using raw lacquer-specific indicators, including laccase retention, viscosity evolution, drying behavior, and final film properties.

### 5.5. Raw Material Variability and Quality Control Perspectives

Source variability, including species, cultivar, growing region, season, tree age, and germplasm background, plays a critical role in raw lacquer quality. Significant variations exist among species and regions in appearance, chemical composition, enzyme activity, moisture content, and film-forming performance [22]. Combined HPLC and ELISA techniques allow for the precise discrimination of lacquer from different sources, revealing, for instance, that small, wood-type lacquer trees have a higher polar urushiol content and smaller glycoprotein molecular weight [1]. This provides a methodological basis for establishing “source–composition–macro performance” correlations and informs cultivar-specific in situ process parameter settings.

**Figure 10:**
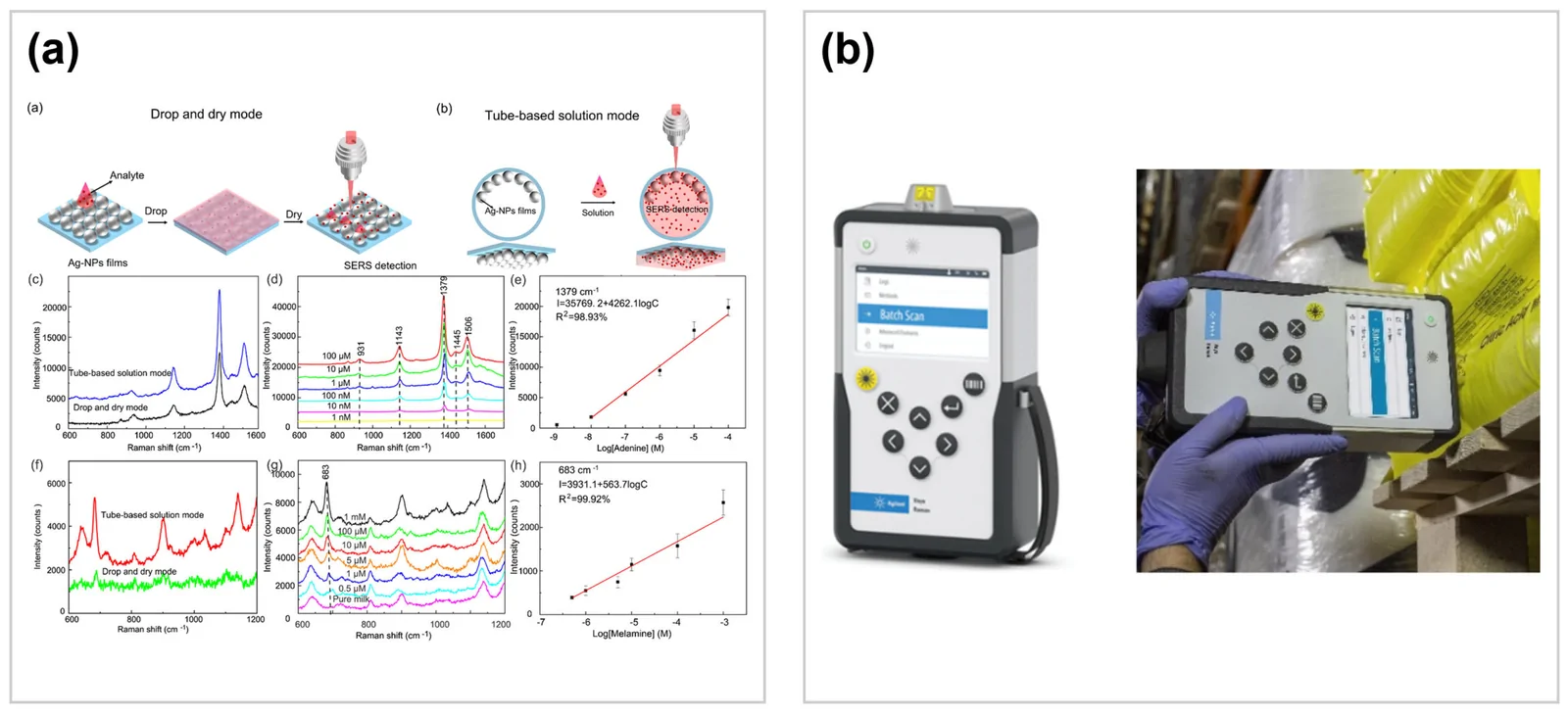
Field-deployable detection and feedback tools for in situ raw lacquer processing: (**a**) tube-based SERS detection workflow, spectral response, and calibration curves for solution analysis; (**b**) examples of portable analytical instruments for on-site inspection and process monitoring. The internal labels within subfigure (**a**) are retained from the adapted source figure [32,36].

Portable analytical devices further support quality monitoring in field processing. Gas chromatography, GC–MS, and optical instruments enable the rapid identification of volatile or characteristic components [32]. Handheld Raman and SERS instruments provide a separate route for rapid detection of urushiol composition and residual impurities [36]. These tools are not substitutes for laboratory analysis; they function best as process monitors for endpoint determination, filtration quality, and conditioning settings. Figure 10 integrates tube-based SERS sensing and portable field instrumentation into a detection–feedback chain [51].

Because raw lacquer is dark, viscous, heterogeneous, and matrix dependent, field SERS, Raman, GC–MS, and optical measurements require calibration transfer, sample-preparation protocols, detection limits, fouling controls, reproducibility checks, and reference laboratory validation before they can support quantitative closed-loop control.

Field detection should provide trend evaluation and anomaly alerts, informing real-time process adjustments based on water content, viscosity, laccase activity, and film properties. Quality-control technologies provide the analytical basis for linking source, harvest season, composition, process parameters, and film performance [40]. This source–composition–process database would enable in situ processing to adjust parameters according to the initial sap condition rather than applying a single fixed recipe.

For decision-making, measured signals should be linked to explicit actions: high moisture should adjust vacuum residence time; high viscosity should trigger lower shear or shorter holding; reduced laccase activity should limit temperature and oxygen exposure; and anomalous Raman/SERS signals should prompt laboratory confirmation before process settings are changed. The suitability of the major in situ processing technologies and their control parameters is compared in Table 8.

## 6. Current Research Gaps and Key Scientific Questions for In Situ Stabilization

Although purification, dehydration, and conditioning technologies provide potential foundations for in situ raw lacquer stabilization, most reported approaches remain at laboratory-scale or conceptual stages. Further field validation is required under variable harvesting conditions, including temperature, humidity, raw material variability, and transportation duration.

### 6.1. Post-Harvest Degradation Kinetics

First, the kinetics of post-harvest sap degradation are still poorly defined. Once sap leaves the tree, exposure to air initiates enzymatic oxidation and polymerization. However, continuous datasets for laccase activity, urushiol composition, viscosity, and film-forming performance across different time, temperature, humidity, impurity load, and storage conditions remain scarce. Without these data, reliable process windows for in situ filtration, dehydration, and temporary storage cannot be established.

### 6.2. Filtration Compatibility and Process Optimization

The relationship between filtration parameters and raw lacquer quality preservation remains insufficiently understood. Although portable filtration devices, centrifugal separation, and advanced membrane concepts provide possible routes for impurity removal, their effects on laccase activity, microemulsion stability, viscosity evolution, and subsequent film performance have not been systematically evaluated. The key scientific question is how filtration parameters, including filtration media characteristics, operating pressure, shear intensity, and exposure time, influence impurity removal while preserving enzymatic activity and coating performance.

### 6.3. Dehydration Process Window

Endpoint control models for in situ dehydration remain insufficient. Excess moisture prolongs drying and reduces storage stability, whereas excessive dehydration shifts rheology and surface drying behavior. The acceptable endpoint is, therefore, a process window rather than a single moisture value. Future studies should establish a multi-parameter dehydration window integrating vacuum level, temperature, residence time, water content, laccase activity retention, viscosity evolution, and subsequent coating performance. Endpoint judgment should integrate solid content, viscosity, laccase activity, surface dry time, gloss, and storage stability rather than relying on water content alone.

### 6.4. Standardized Evaluation and Feedback-Control Framework

Fourth, field-deployed processing still lacks a standardized evaluation system. Conventional criteria target either raw material grading or final film properties; however, in situ processed sap lies between these states. Practical standards should specify impurity limits, moisture range, enzyme activity retention, viscosity range, crusting time, and basic film-forming performance to support equipment comparison, on-site conditioning, and process validation. Such criteria are also needed for inter-site comparison and equipment evaluation. In addition, evidence levels of emerging technologies should be clearly classified to distinguish experimentally validated approaches from conceptual designs.

## 7. Conclusions

Raw lacquer has both cultural significance and engineering potential; however, the traditional dispersed harvesting-centralized processing model causes sap quality loss, longer processing cycles, and product inconsistency. The key issue is how quickly this deterioration can be slowed without sacrificing later curing behavior. This review, therefore, examines potential strategies for mitigating post-harvest deterioration through filtration, vacuum low-temperature dehydration, physical conditioning, and inorganic salt regulation.

In situ processing is better understood as a continuous harvest–purification–dehydration–conditioning–detection workflow than as a substitute technology for traditional refining. By shortening exposure and storage times at the production site, it aims to provide a more stable sap feedstock before downstream refining. Current evidence indicates that portable and positive-pressure centrifugal filtration may improve field impurity removal, while vacuum low-temperature dehydration provides a potential route for water removal under mild thermal conditions. Shear control, oxygen regulation, salt regulation, and enzymatic strategies may further contribute to balancing storage stability and curing capacity. Because raw lacquer remains constrained by viscosity, W/O microemulsion structure, laccase activity, and urushiol oxidation, methods borrowed from other natural product systems, can guide equipment design and parameter selection; however, they still require validation within the lacquer system.

Future validation should proceed in a staged manner: first establishing standardized raw lacquer batches and degradation kinetics, followed by filtration compatibility testing, dehydration-window optimization, and, finally, pilot-scale field validation across cultivars and production regions.

## Figures and Tables

**Table 1:** Scope comparison between this review and earlier lacquer-related reviews.

Review Type	Main Coverage	Gap Addressed Here
Lacquer chemistry and curing reviews	Composition, urushiol oxidation, laccase mechanism, conservation chemistry	Do not organize filtration, dehydration, and field monitoring as a post-harvest process chain
Refining and modification reviews	Film properties, chemical modification, coating applications	Usually address downstream performance rather than stabilization immediately after tapping
This review	Post-harvest filtration, low-temperature dehydration, conditioning, source variability, detection, and control logic	Separates established lacquer evidence from prospective cross-domain technologies and identifies validation gaps

**Table 2:** Composition of evidence in the literature considered in this review.

Evidence Category	Description	Number of Studies
Raw lacquer chemistry and curing studies	Studies directly investigating urushiol, laccase, lacquer composition, oxidation, polymerization, and film formation	26
Raw lacquer refining and processing studies	Studies involving refining, dehydration, filtration, equipment design, and processing parameters	8
Detection and monitoring studies	Studies involving Raman, SERS, portable analysis, and quality evaluation methods	5
Cross-domain analogue studies	Studies from food processing, membrane separation, drying, sensors, or other natural-product fields used only for methodological reference	18

Note: Individual references were categorized according to their primary contribution to this review; some studies may provide evidence relevant to multiple categories. Detailed reference-to-category classification is provided in Supplementary Table S1.

**Table 3:** Comparison between traditional processing and in situ processing of raw lacquer.

Aspect	Traditional Centralized Refining	In Situ Stabilization
Primary objective	Final refining and property adjustment	Early-stage stabilization
Processing stage	After transport	Before transport or before centralized refining
Main challenges	Quality changes during storage and transport	Need for compact equipment and parameter control
Technology maturity	Established	Developing

Note: values are not directly comparable due to different testing conditions.

**Table 4:** Conceptual decision logic for future in situ process optimization.

Measured Signal	Process Stage	Operational Adjustment	Verification Endpoint
High impurity load or declining flux	Filtration	Adjust mesh/media selection, pressure differential, or filter replacement interval	Color change, laccase activity, viscosity, filtrate recovery
Water content above endpoint window	Vacuum dehydration	Increase residence time or adjust vacuum/temperature within enzyme-safe limits	Laccase retention, viscosity rise, surface dry time
Rapid viscosity increases or crusting	Temporary storage/conditioning	Reduce oxygen exposure, lower shear, improve sealing, shorten holding time	Film gloss, hardness, storage stability
Weak drying response after treatment	Final conditioning	Rebalance oxygen exposure, humidity, and mild shear before coating preparation	Surface dry time, practical dry time, film appearance

**Table 5:** Comparison of raw lacquer filtration technologies.

Category	Efficiency and Precision	Main Limitations
Traditional Filtration	Low; fine impurities hard to remove	Low cost but time-consuming
Large Industrial Equipment	High efficiency, good impurity removal	Unfit for field
Portable Filtration Devices	Moderate, faster filtration	Meets lacquer farmers’ needs
Polymeric/Nanosheet Membranes	Tunable pore channels; potential anti-fouling design	Need compatibility tests for viscous W/O lacquer
Centrifugal/Sedimentation Units	Continuous solid–liquid separation; reduced clogging	Require sealing, cleaning, and low-shear operation

**Table 6:** Evidence level and technology maturity of filtration approaches for raw lacquer stabilization.

Technology	Evidence Level	Current Status	Main Validation Requirement
Traditional filtration	Raw lacquer evidence	Established practice	Improve efficiency and reduce air exposure
Portable filtration devices	Engineering evidence	Developing	Field testing, sealing improvement, material compatibility
Positive-pressure centrifugal filtration	Reported lacquer equipment evidence	Requires further validation	Scale-up, cleaning procedure, fouling evaluation, property retention
Polymeric membrane filtration	Cross-domain engineering evidence	Exploratory for raw lacquer	Compatibility with urushiol, laccase retention, fouling behavior
2D nanosheet membranes (GO/MXene/MOFs etc.)	Conceptual/cross-domain evidence	Future research direction	Lacquer-specific filtration tests and safety evaluation

**Table 7:** Conditional comparison of reported raw lacquer film properties under different refining conditions (values should not be directly compared without identical testing protocols).

Measurement/Process Indicator	Raw Lacquer	Traditional Basin Refining (L2 Optimal)	Vacuum-Refined Lacquer	Japanese Ground Pear Lacquer
Moisture Content (%)	30	3–5	3	3.2
Gloss (%)	33	—	98	98
Transmittance (%)	14	Improved	24	25
Viscosity (P)	4.5	High	4.3	4.3
Hardness (7 days)	3H	H	4H	4H
Practical Dry Time (h)	8	2.5	7	6.5
Processing Cycle	—	>7 days	1 day	—
Key Process Parameter Optimization	Baseline	Kurome cycle number, oxygen and hydration control	Vacuum degree, temperature, residence time	Humidity and film performance

**Table 8:** Suitability comparison of key in situ processing technologies for raw lacquer.

Technology	Primary Role	Key Control Parameters	Main Advantages	Main Limitations	In Situ Adaptability
Portable Filtration	Post-harvest impurity removal	Mesh size, flow rate, material compatibility	Simple, suitable for dispersed processing	Difficult to combine high precision and throughput; open filtration prone to crust formation	Suitable as foundational unit; requires sealing and material optimization
Positive-Pressure Centrifugal Filtration	Continuous purification	Pressure differential, centrifugal speed, filter cake	Improves viscous sap throughput, reduces clogging, enables continuity	Complex structure; requires sealing and maintenance	Suitable for engineering upgrades
Vacuum Low-Temperature Dehydration	Concentration and refining	Vacuum, temperature, residence time, endpoint moisture	Protects laccase activity; shortens refining cycles	Miniaturization, energy consumption, and endpoint control require optimization	Core unit of integrated in situ processing
Shear and Oxygen Control	Physical conditioning	Stirring speed, shear rate, oxygen level	Controls viscosity, leveling, and drying performance	Excessive shear may disrupt microemulsion and accelerate inactivation	Coupled with dehydration
Salt/Enzyme Regulation	Storage and curing balance	NaCl, Na_2_CO_3_, pH, enzyme activity	Delays crust formation; regulates curing	Additive safety, residue, and long-term film performance need verification	Suitable for standardized feedstock development
On-Site Quality Detection	Process monitoring	Water content, viscosity, urushiol composition, laccase activity	Provides real-time feedback for filtration, dehydration, and conditioning	Portable detection must be adapted to complex sap systems	Supports intelligent in situ processing

## Data Availability

The original contributions presented in this study are included in the article/Supplementary Materials. Further inquiries can be directed to the corresponding author.

## Supplementary Materials

The following supporting information can be downloaded at: https://www.mdpi.com/article/10.3390/polym18162028/s1, Table S1. Classification matrix of literature sources considered in this review.
