# Supplementary material for: Toward In Situ Stabilization of Raw Chinese Lacquer (Toxicodendron vernicifluum): Current Evidence, Processing Strategies, and Research Challenges
Source: Polymers (Basel). 2026 Aug 21;18(16):2028. doi: 10.3390/polym18162028 (PMC13517471; doi:10.3390/polym18162028)
Supplement: Supplementary file 1 [file polymers-18-02028-s001.zip › polymers-4415343-supplementary.pdf]

Review

Toward In Situ Stabilization of Raw Chinese Lacquer (*Toxicodendron vernicifluum*): Current Evidence, Processing Strategies, and Research Challenges

Ziyue Zhang [1], Baoju Jin [1], Xiaotong Li [1], Hanyun Gao [1], and Xinhao Feng [1,2,*]

Table S1. Classification matrix of literature sources considered in this review

Reference	Raw lacquer chemistry and curing	Raw lacquer refining	Detection and monitoring	Cross-domain analogue	Main contribution
[1]	✓				Links bark anatomy, lacquer yield, and urushiol content among cultivated lacquer-tree varieties.
[2]				✓	Summarizes green pretreatment strategies for quality control of natural products.
[3]				✓	Reviews nanosheet membrane sieving concepts relevant to future filtration design.
[4]				✓	Validates filtration-based dewaterability characterization for complex particulate suspensions under process conditions.
[5]	✓	✓			Examines rapid Chinese lacquer curing mechanisms and related processing technology.
[6]	✓				Defines laccase-mediated urushiol oxidation and film-formation pathways in Japanese lacquer.
[7]	✓				Characterizes enzymatic dehydrogenative polymerization of urushiols in fresh lacquer exudates.
[8]	✓		✓		Tracks oriental lacquer film structure during hardening using analytical characterization.

Reference	Raw lacquer chemistry and curing	Raw lacquer refining	Detection and monitoring	Cross-domain analogue	Main contribution
[9]	✓				Reviews oriental lacquer as a natural polymer and film-forming material.
[10]	✓				Summarizes East Asian lacquer chemistry, art applications, and coating behavior.
[11]	✓				Reviews molecular mechanisms and functional applications of urushiol-based antimicrobial coatings.
[12]	✓	✓			Evaluates repeated Kurome treatment effects on Chinese lacquer and film properties.
[13]	✓				Studies urushi polymerization with epoxidized linseed oil as reactive diluent.
[14]	✓	✓			Investigates naturally drying lacquer hybridized with functional organic silanes under curing conditions.
[15]	✓				Reviews East Asian lacquer chemistry and conservation-oriented scientific literature.
[16]	✓				Explains natural lacquer sap polymerization using special phase-structure mechanisms.
[17]				✓	Optimizes vacuum osmotic dehydration parameters in lettuce processing using response surface methodology.
[18]				✓	Analyzes low-vacuum dehydration conditions for quality retention in sweet pepper.
[19]				✓	Investigates low-temperature vacuum drying behavior in agricultural materials under controlled process conditions.

Reference	Raw lacquer chemistry and curing	Raw lacquer refining	Detection and monitoring	Cross-domain analogue	Main contribution
[20]				✓	Demonstrates low-temperature vacuum drying for preserving papaya functional properties.
[21]				✓	Models mint-leaf drying under varied pressure and temperature conditions.
[22]	✓				Identifies laccol dimer structures and laccase-catalyzed polymerization mechanisms in fresh lacquer sap.
[23]	✓				Reports primary structure of Japanese lacquer-tree laccase as multicopper oxidase.
[24]	✓		✓		Assays China-lacquer laccase activity using microcalorimetric measurement of enzyme reaction behavior.
[25]				✓	Optimizes ultrasound-assisted extraction of phenolic compounds from natural products.
[26]				✓	Evaluates microwave treatment effects on nutritional and bioactive compounds in natural products.
[27]				✓	Demonstrates ultrasound-assisted processing for enhanced Chlorella protein extraction under mild conditions.
[28]				✓	Compares plant-extract and absolute extraction technologies for process selection.
[29]	✓				Optimizes ultrasonic extraction of diene urushiol from lacquer-tree leaves.
[30]				✓	Reviews clean-label extraction of natural bioactives and preservative applications.

Reference	Raw lacquer chemistry and curing	Raw lacquer refining	Detection and monitoring	Cross-domain analogue	Main contribution
[31]				✓	Reviews non-thermal starch modification strategies for polymer-composite applications.
[32]			✓	✓	Reviews portable instruments suitable for on-site environmental sample analysis.
[33]				✓	Demonstrates continuous centrifugal separation and recovery of silver nanoparticles in interfacial systems.
[34]				✓	Studies inclined settlers for continuous solid-liquid separation and washing.
[35]	✓				Examines urushi curing under basic pH using a dual enzyme system.
[36]			✓	✓	Develops tube-based SERS sensing for field-deployable solution detection.
[37]	✓				Identifies laccase as a blue copper oxidase from lacquer-tree latex.
[38]	✓				Reports foundational properties of laccase isolated from lacquer-tree latex.
[39]				✓	Designs thin-film drying equipment and models drying kinetics for whey.
[40]	✓	✓			Reviews modification, processing, and application progress for raw lacquer materials.
[41]				✓	Reviews curing chemistry of air-drying paints as oxidative-polymerization analogue.
[42]	✓				Develops hydrophilic urushiol-based coatings for easy-cleaning oil-pollutant surfaces.

Reference	Raw lacquer chemistry and curing	Raw lacquer refining	Detection and monitoring	Cross-domain analogue	Main contribution
[43]	✓				Synthesizes anisotropic polystyrene/urushiol-lanthanum Janus composite particles with controlled morphology.
[44]				✓	Studies environmentally friendly polybutadiene coatings for corrosion protection under coating design principles.
[45]	✓				Shows silane effects on aging resistance and anticorrosion of natural lacquer.
[46]				✓	Synthesizes radiation-curable hyperbranched resin from epoxy functional fatty acids.
[47]	✓				Prepares tung-oil-modified raw lacquer films for mechanical carving applications.
[48]	✓	✓			Demonstrates copper-ethanolamine nanozymes promoting urushiol oxidation in lacquer curing control.
[49]	✓				Prepares urushiol oligomers and evaluates antibacterial, antioxidant, and thermal stability properties.
[50]	✓				Reconstructs lacquer-tree laccase from protein and copper components to clarify enzyme function.
[51]	✓		✓		Uses pyrolysis GC-MS to study lacquer-film structures and polymerization mechanisms.

Note: A single reference may be assigned to multiple categories when it provides evidence relevant to more than one research aspect.
